# The impact of transparent packaging: how transparent packaging for organic foods affects tourists' green purchasing behavior

**DOI:** 10.3389/fnut.2024.1328596

**Published:** 2024-02-08

**Authors:** TingYue Kuang, Dajun Yang, Dingxia Zou

**Affiliations:** ^1^Faculty of Business, City University of Macau, Macao, Macao SAR, China; ^2^School of Administration, North Sichuan Medical College, Nanchong, Sichuan Province, China

**Keywords:** transparent packaging, organic food, green purchasing behavior, scenic tourism, tourist consumption

## Abstract

Previous studies have shown that transparent packaging can influence consumer behavior, but the impact on tourists' environmentally friendly purchase intentions is not well-understood. This study conducted four experiments with 1,513 participants to explore the role of transparent packaging in tourists' willingness to engage in green purchasing. Factors such as ecological concern, nature connectedness, and environmental consequences were also examined. The results showed that transparent packaging significantly enhanced tourists' purchase intentions and that ecological concern, nature connectedness, and environmental consequences had a significant influence on these intentions. These findings contribute to understanding the packaging paradox and its relationship with tourists' green purchasing behavior. The study has implications for the food retail industry and the promotion of sustainable development in scenic areas, suggesting that transparent packaging can effectively enhance tourists' purchase intentions for green products. Understanding factors like ecological concern and nature connectedness can also provide valuable insights for the industry to improve marketing strategies and promote environmentally friendly choices among tourists.

## 1 Introduction

Food packaging is a source of consumers' perceptions of food (1). In modern food marketing, food packaging affects consumers' perception of interest (2) and purchasing intention (3). The existing researches have the following findings, on the one hand, product packaging increases its attractiveness to consumers. Information about stimulating elements on product packaging, basically the brand logos, designs, and titles (4–6), have the ability to convey information about the products, attract the attention of consumers (7) and affect the consumption expectations of tourists (8). On the other hand, package design can increase the added value of a product (9), and is often applied in sensory marketing. For example, a study by Vasileiadis et al. (10) showed that package design fully satisfies consumers' consumption needs and increases the final value of the product.

Many previous studies have examined the correlation between food packaging and consumer purchase intention, but there is still a lack of research in the realm of tourism food packaging. The existing literature predominantly concentrates on tourists' consumption preferences (11, 12), consumers' sensory perceptions (13), tourism food packaging processes (14), and innovations in food packaging (15). Some studies have paid attention to food packaging transparency (16, 17), but they neglected the impact of food packaging transparency on tourists' green purchasing behavior. Several related studies have shown that food packaging affects tourists' consumer behavior. Pan et al. (18) noticed the impact of green product packaging on consumers' purchase intention; Ho et al. (19) explored how labeling language on food packages affects tourists' purchase intention; Liang et al. (20) demonstrated that small food packages in scenic spots can increase tourists' purchase intention. However, none of the above studies further explored the impact of transparent organic food packaging (vs. opaque) on tourists' green purchasing behavior. As the number of tourists in scenic spots is increasing, their demand for tourists food will increase, too. Therefore, it is of significant research value to further study the impact of transparent organic food packaging (vs. opaque) on tourists' green purchasing behavior in scenic spots.

In recent years, transparent packaging of products has gradually been valued by scholars and they have carried out many relevant researches. Simmonds and Spence (21) have attended to the relationship between food image and transparent packaging. At present, most researches to explore the issue of transparent food packaging mainly focus on topics that related to food safety and quality (11), marketing and consumer behavior (22, 23), packaging design and process (24, 25), as well as the management of tourism and restaurant (26, 27). In the field of packaging design and process, Guzman-Puyol et al. (28) suggested that in order to provide a comprehensive and informative representation, transmittance of different packaging in the visible range should be supplemented with thickness values and graphical images of the analyzed samples. Although the studies of marketing and consumer behavior have focused on the impact of product packaging on its evaluation (29), attractiveness (30, 31) and number of consumed food (32), while the relationship between transparent packaging of organic food and tourist green purchase has been neglected in tourist activities. This study aimed to examine the impact of transparent organic food packaging on tourists' purchase intention, taking into account their ecological concern and nature connectedness. Specifically, it investigated the following research questions: (1) Does transparent packaging (compared to opaque packaging) of organic food in scenic spots positively affect tourists' intention to make environmentally friendly purchases? (2) What is the internal mechanism through which transparent organic food packaging (compared to opaque packaging) affects tourists' eco-friendly purchasing behavior?

In order to fill the gaps of previous studies, this study constructed a research framework, taking organic food packaging (transparent vs. opaque) as the independent variable, while the purchase intention as the dependent variable. Based on ecological concern, environmental consequence, and nature connectedness, this study proposed that transparent organic food packaging improves individuals' environmental perceptions, by enhancing their perception of tourist food quality and safety (33), whereas individuals' ecological concern and environmental consequence increase their concern for tourist food quality and safety (34). Ecological concern mediates the impact of transparent packaging of eco-products on tourists' purchase intention, which means that transparency can increase individuals' purchase intention by enhancing their perception of tourist food quality and safety, thus increasing individuals' environmental attitudes and emotions to promote their purchasing intension (35). First, this study investigated the directly positive impact of food packaging (transparent vs. opaque) on tourists' consumer behavior. Second, from the perspective of ecological concern, this study proposed the mediating role of ecological concern and the moderating role of environmental consequence. The transparent food packaging inspires individuals' care and concern for the ecological environment stimulated by environmental consequence, which will promote their decision-making in purchasing eco-products in the scenic spots. Last, this study demonstrated how the interaction effect between nature connectedness and transparent packaging of organic food affects consumers' purchase intention.

This study made the following contributions to the research on the relationship between organic food packaging (transparent vs. opaque) and tourists' purchase intention. First, although there were common studies on organic food packaging in scenic spots, few have focused on how organic food packaging (transparent vs. opaque) affects tourists' purchase intention. Therefore, this study is an early attempt to explore the transparent packaging of organic food in scenic spots together with tourists' purchase intention, and to discuss their relationship and the internal mechanism of their impacts, which enriched the literature of food packaging, food consumption and tourists' green purchase intention, by empirical research. Second, the mediation model of ecological concern and environmental consequence constructed in this study, enabled better elaboration and theoretical derivation for the internal mechanism with the transparent packaging of product and purchase intention, and revealed the moderating role of environmental consequence. Thirdly, this study identified the moderating effect of environmental consequences on the relationship between eco-product packaging (transparent vs. opaque) and tourists' purchase intention. This further advances the knowledge regarding the association between tourists' environmental awareness and their willingness to engage in green consumption. Fourth, this study identified the interaction effect between nature connectedness and transparent packaging of organic food, further validating that the moderating role of individuals' degree of nature connectedness in the relationship between transparent food packaging and their purchase intention.

In addition, this study is an early attempt to combine transparent organic food packaging with tourists' green purchase intention to discuss whether transparent food packaging would further promote tourists' green purchasing behavior. In other words, this study also expanded the research related to transparent packaging and green purchasing behavior. The main theoretical contribution of this study is the expansion for the impact of organic food transparent packaging in scenic spots, as well as providing a systematic understanding of how it affects tourists' green purchasing behaviors, combined with tourists' individual attributes (ecological concern, environmental consequence, and nature connectedness). The operability and practical significance of this stud, can provide valuable guidance for the sale of organic food in scenic spots, and is helpful for tourists to understand the cultural characteristics of local food and enhance their tourism experience.

## 2 Theoretical background and hypotheses

### 2.1 Transparent packaging paradox

Transparent packaging is made of see-through material that allows the products inside the package to be clearly visible (36), which is characterized by visibility, safety, and enhanced marketing effectiveness. Transparent packaging can be utilized for a variety of products, including food, beverages, cosmetics, and pharmaceuticals, which helps consumers to better understand the materials inside, so that they can make environmentally friendly purchasing decisions (37). Overall, transparent packaging provides much information and choices (38), contributing to a more transparent connection between consumers and manufacturers.

Previous researches on transparent food packaging have primarily examined its impact on food consumption. The inherent characteristics of transparent packaging have given rise to a phenomenon known as the “transparent packaging paradox.” Here are the positive effects it brings about. First, transparent packaging effectively showcases the unique attributes of food products, thereby enticing consumers to make purchases that consequently boost overall consumption (39). On the other hand, since consumers can clearly measure how much food they consume, transparent packaging serves as a monitor and helps to against the overconsumption (40). Consumers can check the material of the food as well as the amount, which enhances the consumer's access to food information on sale and depresses food consumption (36).

Limited researches have been conducted on the impact of transparent packaging for organic food on tourists' purchase intentions, specifically considering their organic food consumption habits. The purchase intention of tourists for organic food with transparent packaging in scenic spots possesses a distinctiveness that sets it apart. First, organic foods are considered to be more beneficial to health and the environment, because they do not involve the pesticides or chemical fertilizers, and haven't been genetically modified (41). So tourists are often more inclined to purchase and supports these foods, due to they can fulfill human needs for health and sustainability. Second, transparent packaging helps tourists visualize food quality. They can learn about the ingredients, sources, and production process of the food through the labels and descriptions on the package, which helps reinforce their trust in organic food (42). Transparent packaging also makes it easier for tourists to follow the food freshness, which is important for consumers who demand high levels of food safety and quality. Last, transparent packaging for organic food not only aligns with the overall environmental atmosphere and the concept of protecting the environment in scenic spots, but also resonates with the idea of sustainability. There is always the emphasis of scenic spots on nature conservation and ecological sustainability, and the transparent packaging of organic food is consistent with the requirements for protecting nature and reducing impacts on the environment. This consistency can increase tourists' interest in organic food and make them more intented on supporting and purchasing these products.

### 2.2 Transparent packaging and tourists' green purchasing behavior

Rowe and Slutzky (43) described two meanings of transparency. The first refers to the property of an object that allows visible light to pass through with less scattering, in which transparency is related to the material and the color of the object itself. The second is the optical property of a figure, which is a spatial order in a wide range. The first physical meaning is favored in product transparency, which is the nature of the product to allow visible light to pass through and scatter less of it, for the purpose of being visible to the human eye. In marketing research, there is a focus on the impact of product packaging on consumer responses. It includes various aspects such as product properties (color, size, volume, and pattern), attention, brand perception, purchase intention, willingness to pay, and consumer behavior (36, 44–49). However, the impact of transparent organic food packaging products on consumers' green purchasing behavior has not received widespread attention.

Transparent packaging is the showcase that provides the outside world with access to the inherent characteristics of a product, realizing the visualization and monitoring of the product (50). Packaging transparency has been categorized into three degrees according to visibility: transparent, translucent and opaque (28). Different degree of packaging transparency have different impacts on consumer attractiveness (51), trust (52), perceived health value (53), environmental friendliness (53), and consumer behavior. The study of Deng and Srinivasan (36) proved the role of transparent packaging on food consumption, which means the increase of food visibility can enhance consumers' purchasing intention. The study of Simmonds and Spence (39) also demonstrated that transparent packaging for smaller food increases the consuming willingness and purchasing intention. The commodity market in the scenic spots is oriented to the needs of consumers, and changing the types, ingredients, packaging, and other elements can create saleable and high-quality products. In this process, transparent packaging of food allows tourists to perceive the quality, ingredients, and content of the food (36), enhancing their trust in the product, satisfying the consumer expectations, and increasing the attraction to consumers, so as to improve consumers' sense of fulfillment in the scenic spots.

However, food selection is a multifaceted process encompassing numerous factors, such as sensory and non-sensory attributes (1). Transparency is related to packaging materials and it can affect consumers' perceptions and evaluations of products. Sabo et al. (51) have compared the attractiveness of healthy and unhealthy food with packages of different degrees of transparency in eight groups. The results found that completely opaque packages were the least attractive and consumers preferred to choose food with transparent packaging. Transparent packaging is considered more instrumental and more aesthetically pleasing than opaque packaging (54). However, the internal mechanism of how transparent packaging (vs. opaque) of organic food affects tourists' green purchasing behavior hasn't been clearly identified. This study aimed to explore tourists' green purchasing behavior toward transparently packaged organic food in scenic spots. Therefore the following hypothesis is proposed.

H1: Transparent (vs. opaque) packaging of organic food can increase tourists' green purchasing behavior.

### 2.3 The mediating role of ecological concern

Ecological concern encompasses both an awareness of environmental issues and a willingness to actively engage in resolving them. It entails a deep understanding of the problems facing our environment and a genuine commitment to taking part in finding solutions. And it also involves individual feelings about green issues and concerns about the depletion of natural resources (55–57). It has been found that there is variability in consumer preferences for product selection (58), which is affected by a number of factors. Those factors have a major impact on tourists' green consumption in scenic spots, mainly education level, age, and gender (59–61). Tourists who care about the environment, as green consumers, tend to adopt green purchasing behavior and purchase green eco-products (59).

Transparent packaging of organic food inspires tourists' ecological concern and enhances their green purchasing behavior. Nowadays, environmental sustainability has gradually become a great concern for people (62). As personal environmental awareness grows, tourists show a greater inclination toward purchasing green and healthy products when visiting scenic spots. Consequently, suppliers in these travel destinations also prioritize sustainable marketing practices. On the one hand, transparent packaging of organic food can enhance tourists' environmental awareness, and their concern for the environment may affect their purchase decision-making of organic food (63). Tourists who are aware of the negative impacts of traditional food process on the environment may be more inclined to choose organic food because the food process is usually more environmentally friendly and sustainable. On the other hand, transparent packaging of organic food can induce health consciousness among tourists, who tend to choose healthy diets, with organic food being believed to be healthier than traditional food (64). Health-conscious tourists may demonstrate a higher inclination to purchase organic food products. So transparent packaging of eco-product in scenic spots can effectively increase the likelihood of eco-product identification for tourist (65), easing their worries and fears about the environment, and greatly increasing their satisfaction (66).

Consequently, we proposed the subsequent hypotheses.

H2: Ecological concern mediates the relationship between transparent packaging (vs. opaque) of eco-products and tourists' green purchasing behavior.

### 2.4 The moderating role of environmental consequences

The correlation between environmental consequences and consumers' purchasing intention is substantial. Environmental consequences are the negative impacts caused by a set of human behaviors to the environment (67). Environmental consequence contributes to the psychological suggestion on consumer's perception of product quality, which can dramatically depress the health index of eco-products, and decrease the demand for that eco-product (68). Findings of researches indicate that consumers' awareness of consequences can exert a favorable influence on their personal norms, consequently shaping their purchase intention (69). Therefore, consumers' purchase intention for green products is higher in a sustainable perspective. There were also studies explored environmental consequence and consumption intention (70), but with transparent product packaging, the environmental consequence of organic food in scenic spots and tourists' ecological concern jointly affect consumers' green purchasing behavior. In summary, transparent packaging (vs. opaque) of ecological products affects tourists' ecological concern, and tourists' purchase intention is affected by the evaluation of ecological products and their own needs, which also triggers tourists' ecological concern in the scenic spots.

Consequently, we proposed the subsequent hypotheses.

H3: The environmental consequences have been found to moderate the relationship between ecological product packaging (transparent vs. opaque) and tourists' green purchasing behavior.

### 2.5 Interaction effect of nature connectedness

Nature connectedness refers to a mental state characterized by perceiving, experiencing, and understanding nature (71). Humans have an innate preference for natural things (72), which is thought to come from biological evolution. This is because natural things are usually safer and more conducive to human survival. Human preference for nature is reflected in various aspects of cognition and behavior, such as aesthetic preferences, moral judgments, and health concepts (73, 74). According to the biophilia hypothesis, the origin of life is closely related to the surrounding natural environment. In 1984, the American biologist Wilsom proposed the biophilia hypothesis, which suggests that over the long course of evolution, human beings have developed a strong tendency to attach themselves to nature and to other beings in the midst of it. From an evolutionary point of view, active adaptation to the natural environment increases the chances of survival for individuals. The internal need for nature connectedness is still retained in modern people and has not faded with industrialization and urbanization (75).

This study concludes that there is an interaction effect between individuals' degree of nature connectedness (high vs. low) and organic food packaging (transparent vs. opaque), and it affects consumers' green purchasing behavior. Consumers are more inclined to purchase eco-friendly products with transparent packaging when they perceive them as natural and organic. Conversely, the influence of packaging transparency on purchase intention is not significant when the product is perceived as man-made. Moreover, when consumers view a product as natural, they tend to connect it with favorable environmental characteristics, such as sustainability, biodegradability, and a low carbon footprint (76). Accordingly, when a product is perceived to be natural and organic, transparent packaging can inform consumers of its natural ingredients and minimal processing, which can enhance consumers' perception of its environmental friendliness (77). Transparent packaging may not have a significant impact on consumers' perception of environmental friendliness when a product was perceived as man-made, as it is less likely to be associated with nature. However, when food in scenic spots is perceived as natural and organic, transparent packaging can enhance tourists' perception of its environmental friendliness and influence their intention to make environmentally friendly purchases. Hence, this study argued that the impact of packaging transparency on tourists' green purchasing behavior for scenic organic food can be moderated by their nature connectedness.

Consequently, we proposed the subsequent hypothesis.

H4: Packaging transparency and nature connectedness have an interaction effect on purchase intention of eco-products.

**Figure 1:**
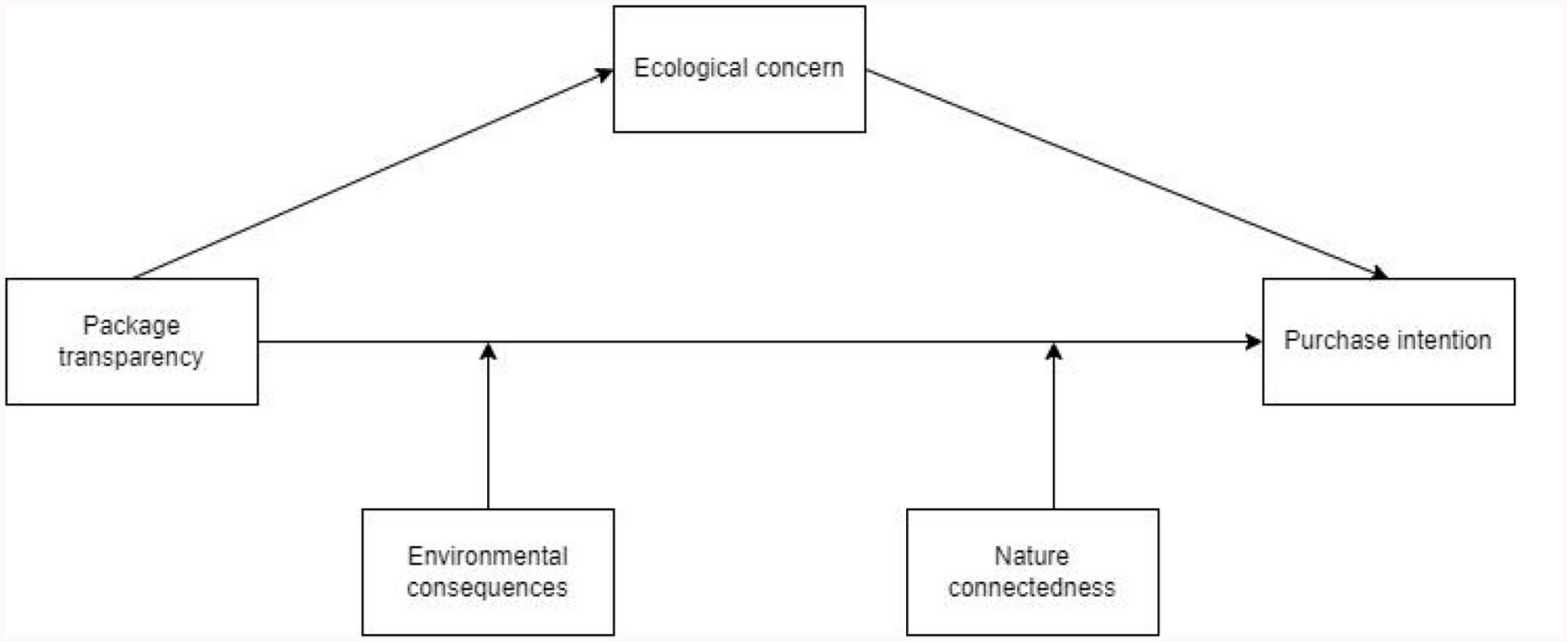
Model concept diagram.

The conceptual model is shown in Figure 1.

## 3 Overview of studies

In this study, three experiments were conducted (Figure 1) in order to validate the four research hypotheses mentioned above. Experiment 1 focused on investigating the impact of transparent packaging (compared to opaque) of organic food on tourists' green purchasing behavior (H1). Experiment 2 aimed to confirm the mediating effect of ecological concern on the relationship between organic food transparent packaging (vs. opaque) and green purchasing intention (H2). Experiment 2 also explored the moderating effect of environmental consequence on the association between ecological concern and purchasing intention (H3). Experiment 3 analyzed the interaction effect between nature connectedness and transparent packaging (vs. opaque) of organic food on tourists' green purchasing behavior (H4). In order to better realize the manipulation of transparent packaging of organic food and the differential impacts of different scenarios on tourists' green purchasing behaviors, different ecological products in the scenic spots were used in different experiments. Ecological rice was used as a stimulus material in Experiment 1, ecological fish was used as a stimulus material in Experiment 2, and organic bread was used as a stimulus material in Experiment 3, which were all packaged in both transparent and opaque packages (see Table 1 for details).

**Table 1:** Research overview.

**Study**	**Study 1**	**Study 2**	**Study 3**	**Study 4**
Purpose	To test for main effects (H1)	To test the mediating role of ecological concerns (H2)	To test the modulation effect of environmental consequences (H3)	To test the interaction effect of nature connectedness (H4)
Independent variable	Organic food packaging (transparent vs. opaque)	Organic food packaging (transparent vs. opaque)	Organic food packaging (transparent vs. opaque)	Organic food packaging (transparent vs. opaque)
Dependent variable	Purchasing intention of eco-products	Purchasing intention of eco-products	Purchasing intention of eco-products	Purchasing intention of eco-products
Methods	ANOVA	ANOVA PROCESS 4	ANOVA PROCESS 1	ANOVA PROCESS 1
Results	Supports H1	Supports H2	Supports H3	Supports H4

## 4 Pilot study

The objective of the Pilot study was to identify appropriate organic foods for the four experiments. A total of 110 participants were randomly recruited from Credamo. To maintain the study's confidentiality, the participants were informed that the chosen products would be applied for a scenic exhibition competition. Subsequently, they were requested to evaluate their intention to purchase six different organic foods. The researcher modified the scale of Sun et al. (78), including the questions of “Do you agree that you are willing to purchase the above food as eco-products in a scenic spots?” (1 = very unwilling, 7 = very willing). The experimental questionnaire contains two groups with total four measurement questions for both transparent and opaque packaging. According to the results of the experiment, ecological rice, ecological fish and organic bread were selected as stimulus materials. In the pilot study, we utilized six organic products, namely ecological rice, ecological fish, organic bread, organic eggs, organic tomatoes, and organic cucumbers, as materials.

## 5 Experiment 1: impact of transparent packaging of organic food on tourists' purchase intention

### 5.1 Experimental design

Experiment 1 conducted a one-way between-group ANOVA (Organic Food Packaging: Transparent vs. Opaque), aiming to investigate the impact of the eco-food transparent packaging in scenic spots on the purchasing intention, so as to verify H1. The researcher recruited 401 participants on the Credamo, of which 202 (50.4%) were male and 199 (49.6%) were female. The age distribution of the participants was 15 (3.7%) under the age of 18, 179 (44.6%) between the ages of 18–25, 131 (32.7%) between the ages of 26–40, 40 (10%) between the ages of 41–60, and 36 (9%) aged 61 years and above. The participants were divided into two groups randomly for the study. In one group, eco-rice was presented with transparent packaging, while the other group received the same eco-rice with opaque packaging. It has been shown that brand, design style and place of origin on the package may affect tourists' decision-making. In order to eliminate their impacts, the eco-rice provided in two groups were with the same package design, while the origin-related information was not disclosed in the product packaging to ensure the effectiveness of manipulation. There were 200 participants in the transparent group (experimental group) and 201 participants in the opaque group (control group). It is worth noting that individuals who have visited tourist attractions within China at least twice per year are eligible to be recruited as participants in our study.

### 5.2 Experimental procedures

**Figure 2:**
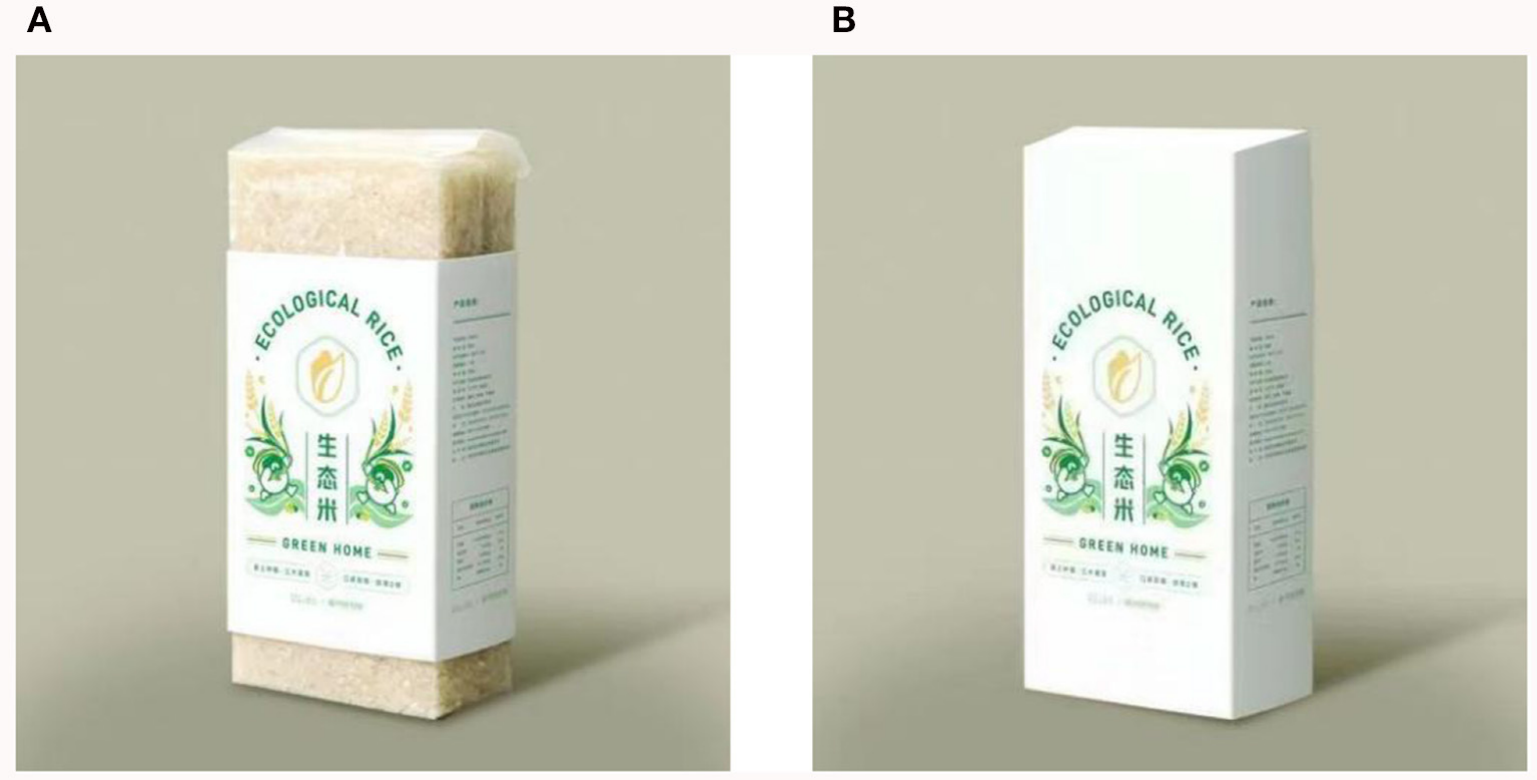
**(A, B)** Experiment 1 stimulus material.

Participants were first asked to read the following contextual information, “Please imagine that you are traveling in a scenic spot where is abundant of ecological rice, and at this time, you are shopping in the tourist shop.” The researchers set up a scenario with a plain scenic spot in breadbasket, took the featured organic food (ecological rice) in transparent packaging as the stimulus material for experimental group, and took the same food (ecological rice) in opaque packaging for control group. Participants then were asked to assess the transparency of the eco-products and their own purchasing intention. The measurement question for transparency was “Many eco-products are sold in the tourist shop, can you see the eco-products in the picture below through the package” (1 = opaque, 2 = transparent). Researchers modified the scale of Sun et al. (78), and guided participants to answer the following measurement question to assess their purchasing intention, “Are you willing to purchase the above product as an ecological agricultural product in the scenic spot?” (1 = very unwilling, 7 = very willing). After the questionnaire was completed, the researchers collected basic demographic information of the participants. The stimulus materials used for the experiment are shown in Figure 2. The relevant questions regarding this experiment have been placed in Table 2.

**Table 2:** Experiment 1 measures the problem.

**Experiment**	**Factors**	**Measurement items**	**Scale source**
Experiment 1	Demographic Information	What is your gender	
		What is your age?	
		What is your education?	
	Packaging transparency	Many healthy eco-products are sold in the scenic area, can you be able to see the eco-products in the picture below through the packaging?	
	Purchase intention	Do you agree that you would like to purchase the above product as a local eco-product of the tourist attraction?	Sun et al. (78)

### 5.3 Experimental results

The researchers conducted tests on the main effects. We used version 27 of the SPSS software as the analysis tool and, under a confidence interval of 95%, studied the relationship between tourists' purchase intention (dependent variable) and the transparency of ecological product packaging (independent variable) through one-way ANOVA. The results indicated that the purchase intention of tourists in the transparent group (M = 5.67, SD = 1.289, SE = 0.091) was significantly higher than that of the opaque group (M = 2.33, SD = 1.73), with a significance level of *P* < 0.05.

In order to test the main effect, researchers took tourists' purchase intention as the dependent variable and the transparency of ecological products packaging as the independent variable to conduct one-way ANOVA, the results showed M_transparent_ = 5.67, SD_transparent_ = 1.289, SE_transparent_ = 0.091, M_opaque_ = 2.33, SD_opaque_ = 1.73, SE_opaque_ = 0.122, F_(1, 399)_ = 477.813, *P* < 0.001, indicating that the independent variables can significantly affect the dependent variable and the purchase intention of the transparent group is higher than that of the opaque group, H1 was verified.

### 5.4 Discussion

Experiment 1 investigated the impact of packaging transparency of ecological products in scenic spots on tourists' purchase intention, thereby validated H1. Our study found that using transparent packaging for eco-products in scenic areas enables tourists to have a more intuitive understanding of the product's quality and ingredients, effectively eliminating the problem of information asymmetry. Transparent packaging allows tourists to fully recognize the green attributes and environmental advantages of the products, stimulating their interest and motivation to make green purchases. In addition, transparent packaging also enhances tourists' credibility and sense of identification with the scenic areas. It provides tourists with a clear and transparent shopping experience, enabling them to make ecological product purchases with greater peace of mind. Tourists perceive that the scenic area advocates environmental protection concepts, prioritizes product quality, and focuses on sustainable development, thus making them more inclined to choose these products. Therefore, the application of transparent packaging in eco-products in scenic areas not only enhances tourists' willingness to make green purchases but also strengthens the brand image of the scenic areas. Our research provides feasible promotional strategies for scenic areas that not only meet environmental requirements but also promote sustainable development, bringing new ideas and driving force to the development of the green tourism industry.

The deficiency of experiment 1 is mainly reflected the fact and fails to analyze the internal mechanism by which the packaging transparency affects tourists' purchase intention of ecological products. Building upon the findings of Experiment 1, Experiment 2 aims to delve into the internal mechanism of how packaging transparency of ecological products in scenic spots affects tourists' purchase intention. Additionally, this study would investigate the mediating role of ecological concern and the moderating role of environmental consequence in this relationship.

## 6 Experiment 2: the mediating role of ecological concern

### 6.1 Experimental design

Experiment 2 was conducted, (1) to verify the main effect for the impact of eco-products packaging transparency on consumers' purchase intention, (2) to verify the mediating role of ecological concern in impact of eco-products packaging transparency in the scenic spots on tourists' purchasing intention, and (3) to verify that the moderating role of environmental consequences in the mediation of ecological concern for impact of eco-products packaging transparency on tourists' purchasing intention. For experiment 2, ecological fish was selected as the stimulus material. The addition of information about the product brand—“Qianfu Fish” on the product package and the same product design for both experimental group and control groups can effectively reduce its impact on the relationship between package transparency and consumers' purchasing intention.

In this study, a one-way between-groups analysis of variance (ANOVA) was performed to compare the effects of different product packaging (transparent vs. opaque). A total of 406 participants were recruited from Credamo, and one incomplete questionnaire was excluded, resulting in a final valid sample size of 405 participants. Of the participants, 201 (49.6%) were males, and 204 (50.4%) were females. The age distribution indicated that 20 (4.9%) were below 18 years old, 183 (45.2%) were between 18–25 years old, 177 (43.7%) were between 26–40 years old, 15 (3.7%) were between 41–60 years old, and 10 (2.5%) were 61 years old and above. The participants were randomly assigned to two groups. The experimental group consisted of 203 participants who received eco-products in transparent packaging, while the control group included 202 participants who received eco-products in opaque packaging. It is worth noting that individuals who have visited tourist attractions within China at least twice per year are eligible to be recruited as participants in our study.

### 6.2 Experimental procedure

**Figure 3:**
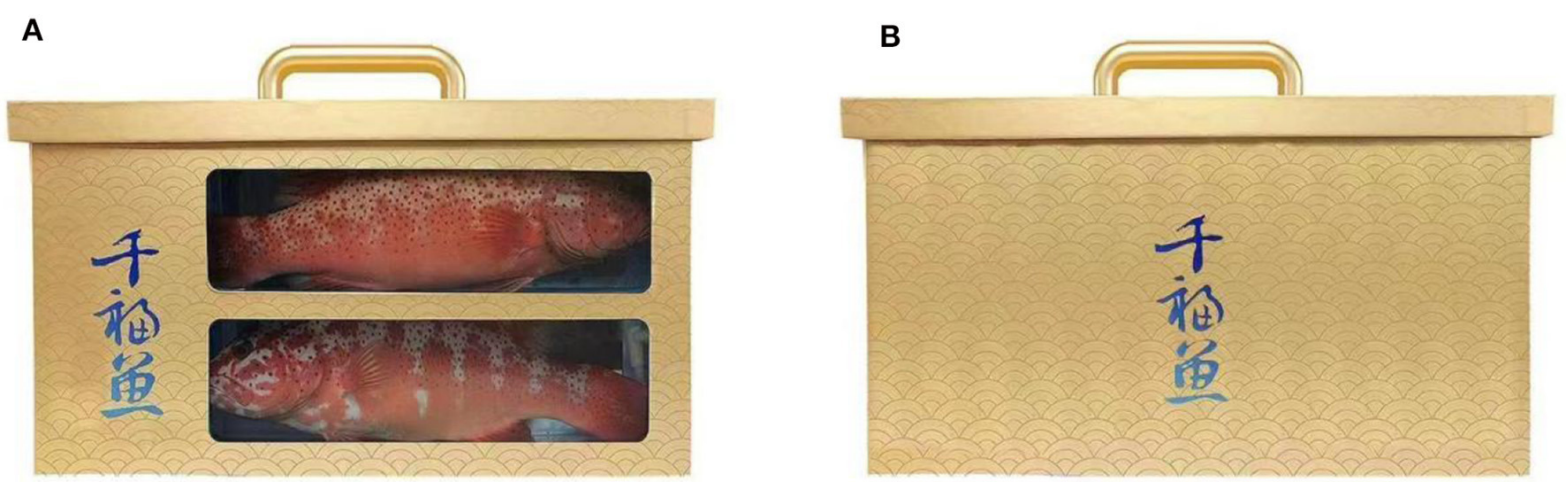
**(A, B)** Experiment 2 stimulus material.

Participants were guided to imagine themselves traveling around a scenic lake area where a tourist shop offers eco-products for sale. Participants then were asked to assess the transparency of the eco-products and their own purchasing intention. The measurement question for transparency was “Many eco-products are sold in the tourist shop, can you see the eco-products in the picture below through the package” (1 = opaque, 2 = transparent). The researchers then adapted the Environmental Concern Scale from Dunlap et al. (79) to assess participants' ecological concern with a total of three questions, including “Do you agree that the more transparent the packaging of an eco-product is, the more you can feel its contribution to ecological conservation, and thus reduces your negative feelings about environmental damage?”, “Do you agree that the more transparent the packaging of an eco-product, the more you can feel about its contribution to environmental protection?”, and “Do you agree that the more transparent the packaging of an eco-product, the more you can feel its efforts toward the natural balance?” (1 = strongly disagree, 7 = strongly agree). Meanwhile, the researchers modified the scale of Follows and Jobber (67) to assess the participants' perception of environmental consequence with a total of three questions, such as “Do you agree that the impact of eco-products on environmental protection is very important to you?” (1 = strongly disagree, 7 = strongly agree). Researchers also assessed their purchasing intention with the question, “Are you willing to purchase the above product as an ecological agricultural product in the scenic spot?” (1 = very unwilling, 7 = very willing). After the questionnaire was completed, the researchers collected basic demographic information of the participants. The stimulus materials used for the experiment are shown in Figure 3. The relevant questions regarding this experiment have been placed in Table 3.

**Table 3:** Experiment 2 measures the problem.

**Experiment**	**Factors**	**Measurement items**	**Scale source**
Experiment 2	Demographic information	What is your gender?	
		What is your age?	
		What is your education?	
	Packaging transparency	Many healthy eco-products are sold in the scenic area, can you be able to see the eco-products in the picture below through the packaging?	
	Ecological concern	Do you agree that the more transparent the packaging of an eco-product is, the more it will make you feel that the product is contributing to ecological preservation, thus reducing your negative feelings about environmental damage?	Dunlap et al. (79)
		Do you agree that the more transparent the packaging of an eco-product is, the more it enhances your recognition of the product's contribution to environmental protection?	
		Do you agree that the more transparent the packaging of an eco-product is, the more it makes you feel that the product is making an effort to balance nature?	
	Purchase intention	Do you agree that you would like to purchase the above product as a local eco-product of the tourist attraction?	Sun et al. (78)

### 6.3 Experimental result

In this study, researchers conducted a test to examine the main effect. We used SPSS software version 27 as an analytical tool with confidence intervals set at 95%. They utilized a one-way ANOVA to analyze the relationship between tourists' purchase intention and the transparency of ecological product packaging, considering purchase intention as the dependent variable and the transparency as the independent variable. The results revealed that the purchase intention for ecological products was significantly greater in the experimental group (M = 6.54, SD = 1.271, SE = 0.089) compared to the control group [M = 2.63, SD = 0.769, SE = 0.054, F_(1, 403)_ = 1,395.861, *P* = 0.000].

**Figure 4:**
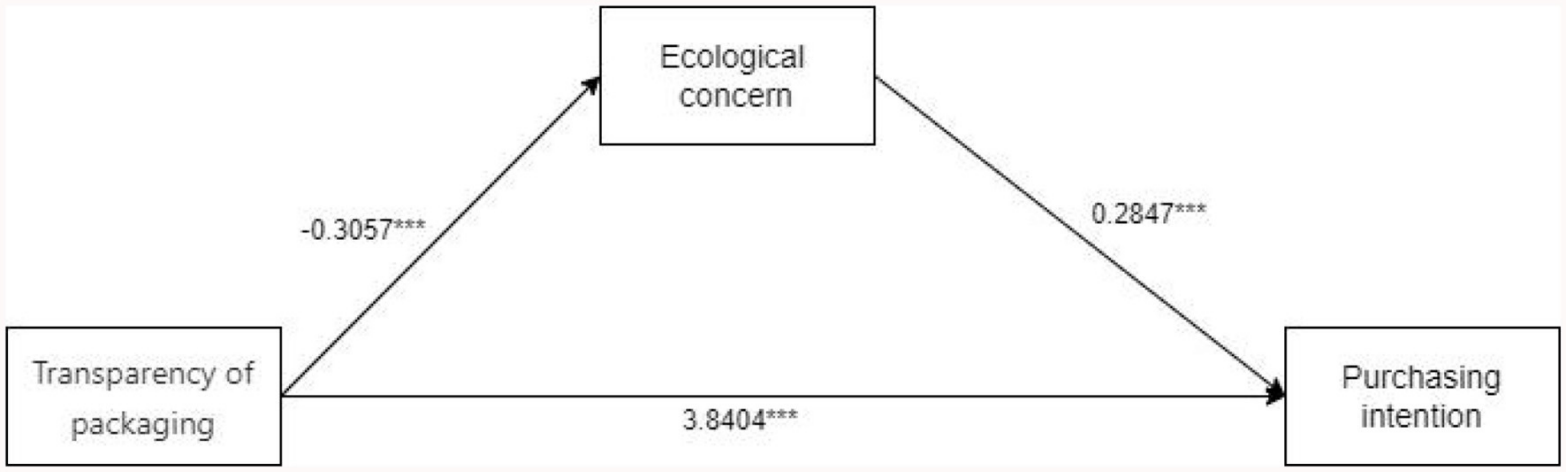
Experiment 2 results of mediating effects.

In this study, a mediation analysis was performed to investigate the relationship between transparency of organic food packaging, ecological concern, and tourists' purchasing intention of eco-products. The study also included education level and gender as control variables. The mediating effect of ecological concern on the impact of eco-product packaging transparency in scenic spots was analyzed using Process model 4 [Bootstrap sample: 5,000; (80)]. The results show that the mediating effect for packaging transparency of eco-products-ecological concern-purchasing intention of eco-products is significant (β = 3.7534, SE = 0.1083, 95% CI [3.5405–3.9663]). The coefficient of ecological concern-purchasing intention of eco-products is 0.2847^***^, and packaging transparency coefficient of ecological products-purchasing intention of ecological products is 3.8404^***^. Therefore, ecological concern is fully mediating between the transparency of ecological products in scenic spots and the purchasing intention of ecological products. H2 was verified. The experimental results are shown in Figure 4. H2 was verified.

### 6.4 Discussion

The research findings from experiment 2 revealed that the relationship between transparent packaging of eco-products and tourists' green purchase intention is mediated by their concern for ecological issues. Transparent packaging enables a more intuitive display of product quality and ingredients, thereby enhancing tourists' recognition and trust in the products, and stimulating their green purchase intention. Our study uncovers the mechanism through which transparent packaging enhances tourists' green purchase intention, namely by influencing their purchase decisions through concern for ecological issues. This finding holds practical significance for scenic areas and eco-product suppliers, providing them with an effective strategy for promoting green purchases. Furthermore, our study offers new perspectives and foundations for relevant theories and empirical research.

## 7 Experiment 3: the moderating role of environmental consequences

### 7.1 Experimental design

The purpose of Experiment 4 is to explore the regulatory effect of environmental consequences on packaging transparency. We designed a single-factor two-level variance experiment (ecological product packaging transparency: transparent vs. opaque) X2 (environmental consequences: high vs. low) in Experiment 3. The experiment aims to verify the main effect of packaging transparency of ecological products on consumers' purchase intentions and discuss the moderating effect of environmental consequences on packaging transparency of ecological products and tourists' purchase intentions (H). We randomly recruited 300 participants on the Credamo platform, including 151 males (50.3%) and 149 females (49.7%). The age distribution of the participants is as follows: below 18 years old (3.3%), 18–25 years old (27%), 26–40 years old (28%), 41–60 years old (20.3%), and above 61 years old (21.3%). All participants were randomly assigned to two scenarios, one with transparent packaging for ecological products and the other with opaque packaging. The transparent group (manipulation group) consisted of 151 participants, while the non-transparent group (control group) consisted of 149 participants. It is worth noting that individuals who have visited tourist attractions within China at least twice per year are eligible to be recruited as participants in our study.

### 7.2 Experimental procedures

**Figure 5:**
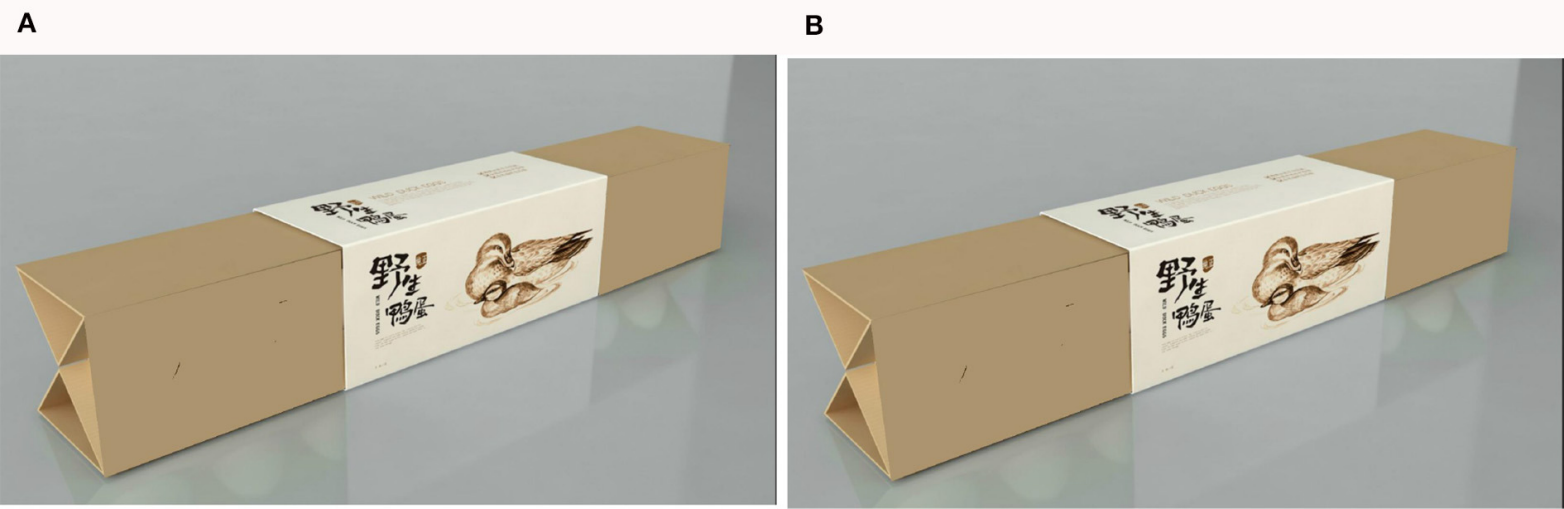
**(A, B)** Experiment 3 stimulus material.

First, we asked all participants to imagine themselves traveling in a mountainous scenic area where there is a large shopping mall. At this moment, you are browsing the eco-friendly products being sold by the staff at the mall. In the transparent condition, transparent packaging of eco-friendly duck eggs is used as the stimulus material in Experiment 3. In the opaque condition, we used packaging that is non-transparent but identical in content to the eco-friendly products. Then, participants were asked, “In the scenic area, there are many healthy eco-friendly products for sale. Can you see the eco-friendly products depicted in the image below through the packaging?” (1 = opaque, 2 = transparent), to assess the transparency of the packaging of eco-friendly products. Participants needed to respond to the following questions: “Do you agree that the environmental impact of eco-friendly products is important to you?”, “Do you agree that eco-friendly food will significantly affect the waste in landfills?”, and “Do you agree that eco-friendly products can effectively alleviate resource depletion?” These three questions were used to measure the environmental consequences of the participants' responses (1 = strongly disagree, 7 = strongly agree) (67). Then participants were asked, “Would you be willing to purchase the aforementioned products as locally produced eco-farming products in the tourist scenic area?” (1 = very willing, 7 = very unwilling) (Cronbach's α = 0.648). The stimulus materials used for the experiment are shown in Figure 5. The relevant questions regarding this experiment have been placed in Table 4.

**Table 4:** Experiment 3 measures the problem.

**Experiment**	**Factors**	**Measurement items**	**Scale source**
Experiment 3	Demographic Information	What is your gender?	
		What is your age?	
		What is your education?	
	Packaging transparency	Many healthy eco-products are sold in the scenic area, can you be able to see the eco-products in the picture below through the packaging?	
	Environmental consequences	Do you agree that the impact of eco-products on the environment is important to you?	Follows and Jobber (67)
		Do you agree that eco-food adds to landfill waste is important to you?
		Do you agree that eco-products can be effective in curbing some resource depletion?
	Purchase intention	Do you agree that you would like to purchase the above product as a local eco-product of the tourist attraction?	Sun et al. (78)

Consistent with Experiment 2, considering that gender may influence tourists' perception of and willingness to purchase ecotourism products, we controlled for gender in Experiment 3. Finally, we collected demographic information related to the participants.

### 7.3 Experimental results

Main effect analysis. We used SPSS software version 27 as an analytical tool with confidence intervals set at 95%. We conducted a one-way analysis of variance with the willingness to purchase ecological products as the dependent variable and the transparency of ecological product packaging within the scenic area as the independent variable. The experimental results showed: M transparent group = 6.13, SD transparent group = 1.615, SE transparent group = 0.131; M opaque group = 4.74, SD opaque group = 1.242, SE opaque group = 0.102; F_(1, 298)_ = 69.432, *P* < 0.001. Therefore, it can be concluded that the willingness to purchase ecological products with transparent packaging is significantly higher than that of the opaque group, indicating a significant impact of packaging transparency on tourists' willingness to purchase ecological products.

**Figure 6:**
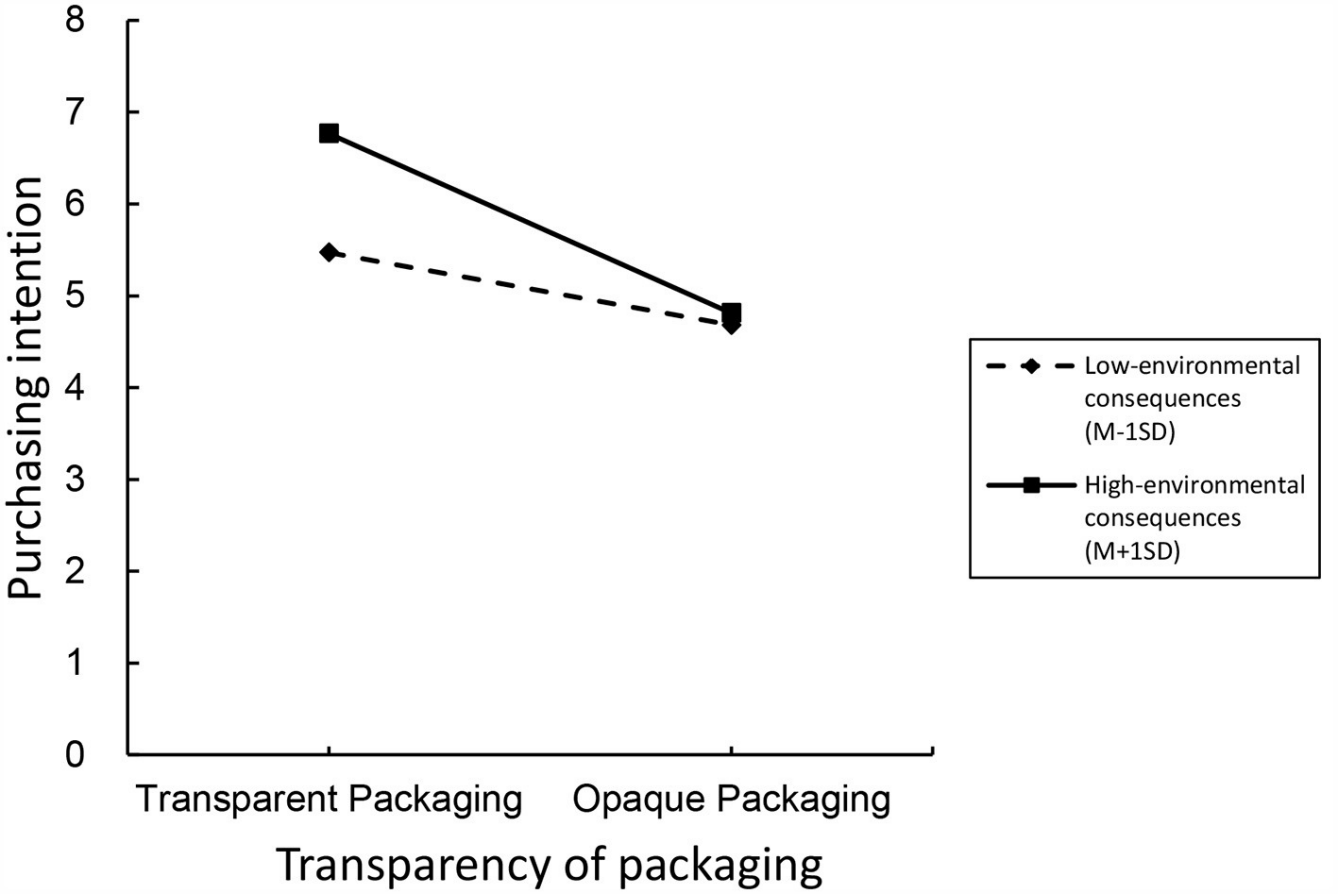
Experiment 3 moderating effect results.

Moderation effect analysis. We used the willingness to purchase ecological products as the dependent variable, the transparency of ecological product packaging within the scenic area as the independent variable, and environmental outcomes as the moderator variable. We attempted to verify the interaction effect of environmental outcomes and packaging transparency on tourists' willingness to purchase ecological products. We used Process Model 1 to test the moderation effect of environmental outcomes [Bootstrap sample: 5,000, (80)]. The experimental results showed that the main effect of packaging transparency on tourists' willingness to purchase ecological products was significant (β = −1.3729, *P* < 0.001), the main effect of environmental outcomes on tourists' willingness to purchase ecological products was significant (β = 0.4669, p < 0.001), and the interaction effect between environmental outcomes and ecological product packaging transparency on tourists' willingness to purchase ecological products was significant (β = −0.7559, *P* < 0.001). The experimental results were placed in Figure 6.

Control variable test. We used gender as the independent variable and the willingness to purchase ecological products as the dependent variable to conduct a one-way analysis of variance. The experimental results showed that gender had no significant effect on tourists' willingness to purchase ecological products [F_(1, 298)_ = 0.259, *P* = 0.611]. Therefore, alternative explanations related to the control variable of gender are excluded, and hypothesis H1 is further validated.

### 7.4 Discussion

In Experiment 3, we confirmed the significant role of ecological product packaging transparency in influencing tourists' purchase intention, thus validating hypothesis H1. We also found evidence for the moderating effect of environmental consequences on the relationship between ecological product packaging transparency and tourists' purchase intention. The results indicate that under conditions of high environmental consequences and transparent packaging, tourists have the highest purchase intention for ecological products. On the other hand, under conditions of low environmental consequences and opaque packaging, tourists' purchase intention is relatively lower. Additionally, the results showed a declining trend in tourists' purchase intention under conditions of high environmental consequences and opaque packaging.

## 8 Experiment 4: interaction effect of nature connectedness

### 8.1 Experimental design

Experiment 4 was conducted, (1) to verify the main effect of packaging transparency of ecological products on consumers' purchasing intention (H1), and (2) to discuss the moderating effect of nature connectedness on the packaging transparency of ecological products in scenic spots and tourists' purchasing intention from the perspective of tourists' environmental behavior (H4). In Experiment 4, organic bread made from ecological wheat was selected as the stimulus material, which removed all the brand information of the product on the package to reduce its influence on the participants' consumer behavior.

The researchers designed a one-way between-groups ANOVA for (Transparency of eco-product packaging: transparent vs. opaque) X 2 (Nature connectedness: high vs. low). Four hundred and seven participants were recruited at the Credamo, of which 203 (49.9%) were male and 204 (50.1%) were female. The age distribution of the participants was 13 (3.2%) under the age of 18 years, 111 (27.3%) between the ages of 18–25 years, 256 (62.9%) between the ages of 26–40 years, 13 (3.2%) between the ages of 41–60 years, and 14 (3.4%) aged 61 years and above. The participants were randomly divided into two groups, in the scenario of one group presenting eco-product with transparent packaging, and the other group with opaque packaging. There were 204 participants in the experimental group with eco-product in the transparent packaging, and 203 participants in the control group with eco-product in the opaque packaging. It is worth noting that individuals who have visited tourist attractions within China at least twice per year are eligible to be recruited as participants in our study.

### 8.2 Experimental procedures

**Figure 7:**
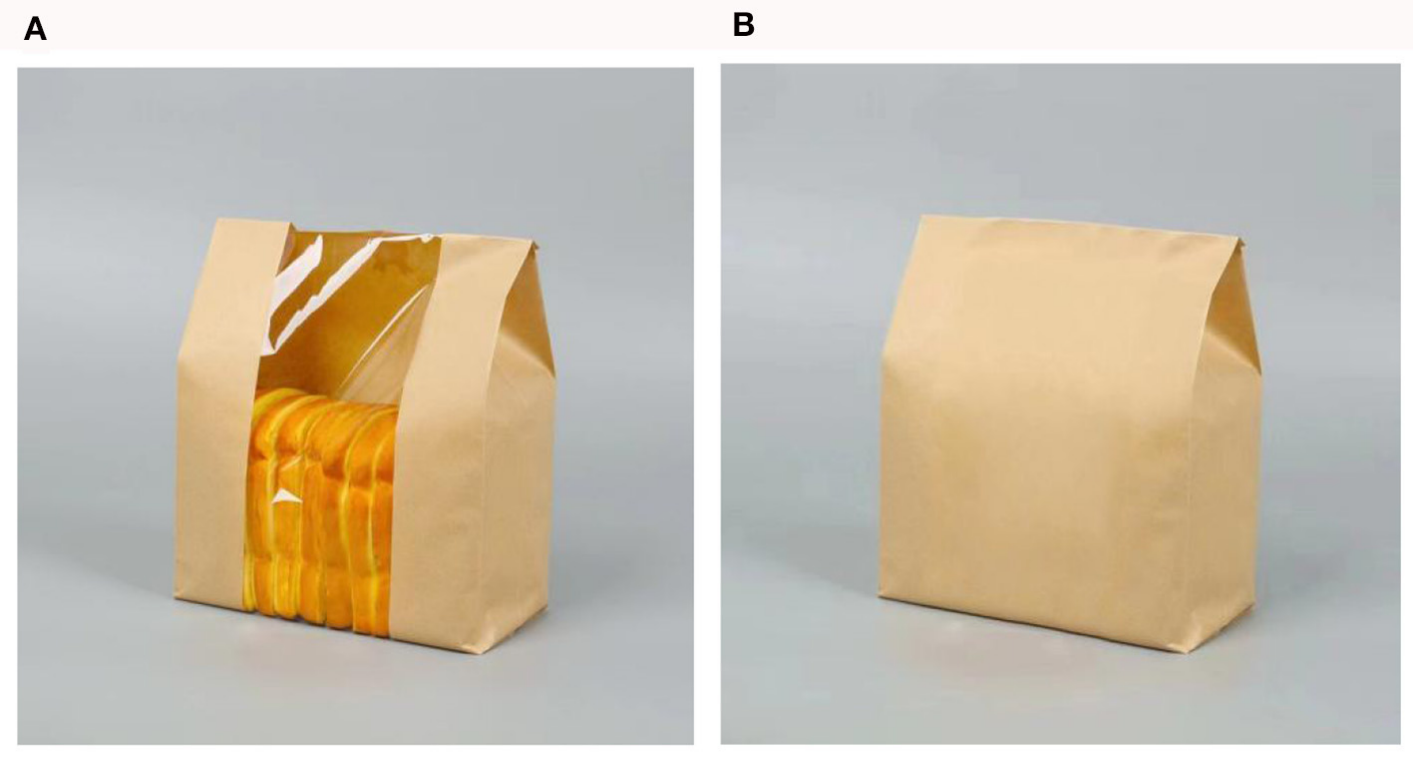
**(A, B)** Experiment 4 stimulus material.

Participants were guided to imagine themselves embarking on a journey through a breathtaking scenic area characterized by rolling hills. Within this captivating landscape, there exists a charming tourist shop that offers a range of eco-friendly products. In this experiment, the organic bread selected from the pilot study with both transparent and opaque packaging were took as the stimulus material. Participants then were asked to assess the transparency of the eco-products. The measurement question for transparency was “Many eco-products are sold in the tourist shop, can you see the eco-products in the picture below through the package” (1 = opaque, 2 = transparent). The researchers adapted the Nature Connectedness Scale developed by Mayer and Frantz (81) to measure participants' nature connectedness with questions such as, “Do you agree that the natural world is a community to which you belong?,” “Do you agree that when you think about your life, you can imagine yourself as a part of a larger life cycle?,” and “Do you agree that when you are in a scenic spot, you feel at one with the nature around you?” (1 = strongly disagree, 7 = strongly agree). Researchers also assessed their purchasing intention with the question, “Are you willing to purchase the above product as an ecological agricultural product in the scenic spot?” (1 = very unwilling, 7 = very willing). After the questionnaire was completed, the researchers collected basic demographic information of the participants. The stimulus materials used for the experiment are shown in Figure 7. The relevant questions regarding this experiment have been placed in Table 5.

**Table 5:** Experiment 4 measures the problem.

**Experiment**	**Factors**	**Measurement items**	**Scale source**
Experiment 4	Demographic Information	What is your gender	
		What is your age?	
		What is your education?	
	Packaging transparency	Many healthy eco-products are sold in the scenic area, can you be able to see the eco-products in the picture below through the packaging?	
	Natural connection	Do you agree that nature is a community to which I belong?	Mayer and Frantz (81)
		Do you agree that when you think about your life, you can visualize yourself as part of a larger cyclical process of life?	
		Do you agree that when you are in a scenic area, you feel at one with the natural world around you?	
	Purchase intention	Do you agree that you would like to purchase the above product as a local eco-product of the tourist attraction?	Sun et al. (78)

### 8.3 Experimental results

In order to examine the main effect, a test was conducted by the researchers. We used SPSS software version 27 as an analytical tool with confidence intervals set at 95%. The study used one-way ANOVA to analyze the relationship between tourists' purchase intention of eco-products (dependent variable) and the transparency of ecological product packaging (independent variable). The results showed that the purchase intention of ecological products in the experimental group (M = 6.17, SD = 1.154, SE = 0.081) was significantly higher than that of the control group [M = 1.76, SD = 1.76, SE = 0.071, F_(1, 405)_ = 1,682.013, *P* < 0.001].

**Figure 8:**
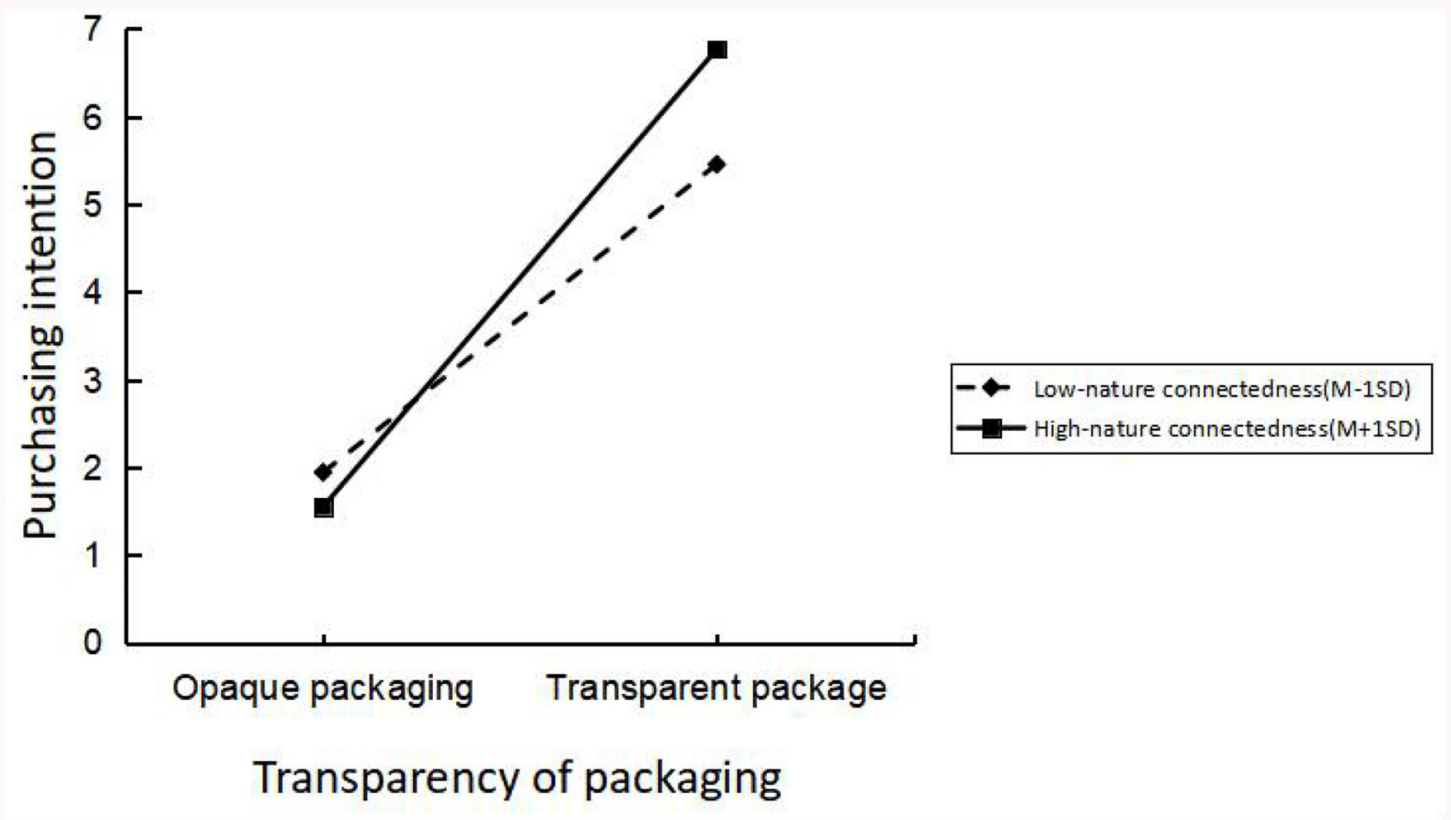
Experiment 4 moderating effect results.

The researchers conducted an analysis to examine the interaction effect of packaging transparency and nature connectedness on tourists' purchasing intention for ecological products in scenic spots. The outcome variable was measured. A two-way ANOVA was performed with two factors: ecological product packaging transparency (transparent vs. opaque) and nature connectedness (high vs. low). The results revealed significant main effects for both packaging transparency and nature connectedness on tourists' purchasing intention [F_(1, 407)_ = 1,609.352, p = 0.000; F_(15, 391)_ = 3.759, *p* = 0.000, respectively]. Additionally, a significant interaction effect was observed between nature connectedness and the transparency of ecological product packaging [F_(16, 390)_ = 118.606, *p* = 0.000]. Therefore, H4 was validated. The experimental results were placed in Figure 8.

### 8.4 Discussion

Our experiment revealed that nature connectedness moderates the relationship between transparent packaging of ecological products (vs. opaque) and tourists' willingness to purchase green products. Notably, we contribute by providing empirical evidence showing that individuals with higher levels of nature connectedness are more likely to be influenced by transparent packaging, exhibiting a greater inclination toward purchasing ecological products. This finding underscores the importance of considering individuals' psychological connection with nature in designing effective packaging strategies to promote green consumption. Furthermore, our study sheds light on the potential role of nature connectedness as a key factor in driving sustainable consumer behavior. Future research should focus on exploring additional factors that may impact green purchase intentions and encompass a wider range of consumer samples to enhance the generalizability of our findings.

## 9 General discussion

### 9.1 Conclusions

This study explored the impact of organic food packaging (transparent vs. opaque) on consumers' purchasing intention in scenic spots. By manipulating organic food packaging (transparent vs. opaque) and different samples of participants, it can be found that tourists trust organic food with transparent packaging more. Experiment 1 showed that packaging (vs. opaque packaging) of organic food in scenic spots was more able to inspire tourists' purchasing intention, which validated H1. Experiment 2 showed that ecological concern mediated the relationship between transparent packaging (vs. opaque) of organic food in scenic spots and tourists' purchasing intention, which validated H2. The further research showed that environmental consequence as a moderating variable could effectively moderating the relationship between ecological concern and purchasing intention, which validated H3. The results of Experiment 3 showed that the degree of nature connectedness of individual tourists and the packaging of ecological products (transparent vs. opaque) had a significant interaction effect on tourists' purchasing intention, which validated H4.

In general, this study explores the impact of organic food packaging (transparent and opaque) on consumers' purchase intention in tourist areas, revealing that transparent packaging can stimulate tourists' purchase intention more effectively. At the same time, the study finds that ecological concern plays a mediating role in the relationship between transparent packaging of organic food in tourist areas and purchase intention, and environmental consequences serve as a moderating variable in the relationship between ecological concern and purchase intention. Furthermore, tourists' level of connection with nature also has a significant interactive effect on the relationship between packaging transparency and purchase intention. These findings hold important theoretical implications for the design and marketing strategies of organic food packaging in tourist areas, providing guidance for further boosting the market development of organic food.

### 9.2 Theoretical implication

This study has the following theoretical implications. First, although many studies have focused on the relationship between food packaging and purchasing intention (20, 82), few studies have focused on the impact of organic food packaging (transparent vs. opaque) in scenic spots on tourists' purchasing intention. Although the packaging of organic food has been identified as an important factor affecting the consumer behavior (42), its impact on tourists' consumer behavior has not been fully investigated. In response to recent research callings (83, 84) for greater attention to the impacts of food packaging on tourists (85), this study examined the influence of transparent organic food packaging in scenic spots from the perspective of ecological concern and nature connectedness. On the one hand, this study extends the literature on tourists' pro-environmental behaviors, and on the other hand, it found transparent packaging (vs. opaque) of organic products is more effective in stimulating tourists' ecological concern. The results of this study can help to better understand the impact of transparent organic food packaging in scenic spots on tourists' green purchasing behavior.

This study provided new insights based on the impact of transparent packaging of organic food on tourists' green purchasing behavior. The impact of transparent packaging of organic food could be evaluated by the interaction effect between tourists' degree of nature connectedness and their green purchasing behavior. Previous studies have confirmed that transparent packaging highlights the physical characteristics of the product (86), which affects consumers' judgment (87). Related studies on transparent packaging and purchasing intention have not revealed how organic food with transparent packaging affects consumers' purchasing intention from the perspective of the difference in individual preference to nature. This study suggested that the purchasing intention of tourists with a high degree of nature connectedness can be more easily motivated. Therefore, this study extended the literature related to transparent packaging of organic food and tourists' green purchasing behavior, and also provides a new perspective for research on the retailing of organic food. The truth is with the impact of transparent packaging of organic food, individuals who are closer to nature may pay more attention to the products with transparent packaging.

The relationship between environmentally consequences of transparent packaging (transparent vs. opaque) for organic food and tourists' green purchasing intentions is explored in this study. It suggests that transparent packaging allows consumers to see the food inside the packaging clearly, enabling them to assess the quality, freshness, and compliance with organic standards. More importantly, transparent packaging provides consumers with transparency regarding the food, fostering trust in the product, and increasing purchasing intentions. As demonstrated by Sabri et al. (50), transparent packaging of ecological products may to some extent signal the quality of the product and the environmental attributes of the packaging material to tourists. Consumers tend to prefer purchasing products that they can see, as transparent packaging reassures them about food safety. It is worth noting that transparent packaging is generally made of plastic, and the production and disposal processes of plastic have certain environmental impacts (88). The production of plastic requires non-renewable resources such as petroleum and involves significant emissions of greenhouse gases and pollutants. Therefore, the environmental consequences of transparent packaging materials can effectively influence tourists' green purchasing intentions.

### 9.3 Managerial implication

For one thing, the results of this study provided valuable suggestions for organic food retailing in scenic spots. The three experiments in this study consistently found that transparent packaging alone for organic food sold in scenic spots was able to stimulate tourists' green purchasing intention. Therefore, organic food retailers in scenic spots should consider adopting transparent packaging when selling organic food so that it can help to enhance tourists' purchasing intention for their products. Furthermore, in Chinese scenic spots, retailers can try to sell organic food in transparent packaging so as to protect the environment and increase retail sales.

For another, this study also found that individual-level attributes such as tourists' ecological concern and nature connectedness mediated the relationship between transparent packaging and organic food purchasing intention. Based on this, on the one hand, managers of scenic spots can adopt activities that stimulate tourists' ecological concern and nature connectedness to arouse their pro-life awareness, and on the other hand, to increase retail sales of organic food. Specifically, only those products that can generate ecological concern as well as nature connectedness among tourists can effectively activate their green purchasing behavior. This study suggested that organic food retailers should pay more attention to the impact of transparent packaging of organic food on tourists' purchasing behavior, and try to build a trust for tourists on the transparent organic food packaging.

### 9.4 Limitations and future research

There are certain limitations to consider in this study. Primarily, its external validity is limited. In order to strengthen external validity, future research could incorporate more field research, rather than solely relying on virtual scenarios used in all three experiments of this study to enhance internal validity (89). Second, this study examined the interaction effect of ecological product packaging transparency in scenic spots and nature connectedness on tourists' purchasing intention. The experimental results showed that the higher the transparency of ecological product packaging in scenic spots, the higher degree of nature connectedness, and could enhances the tourists' purchasing intention. Based on this study, it can be concluded that tourists who value the natural environment prefer packaging that enables them to have a clear view of the product contents. However, it is worth noting that the influence of translucent packaging on consumer behavior has not been thoroughly examined. Future research should focus on investigating the potential effect of translucent packaging on consumers' purchasing intentions. Third, this study focused solely on the moderating effect of environmental consequences and nature connectedness, which are both outcomes of tourists' individual knowledge and comprehension of the external environment. Tourists' subjective cognitive cultivation, knowledge reserve, status, and so on, as well as objective product attributes, product brands, and business environments may also be potentially influencing factors. Therefore, this study poses the following question, “What is the interaction effect between transparent product packaging and consumption environment in the scenic spots?” and “Does tourists' place attachment moderate the relationship between product packaging transparency and tourists' purchasing intention?,” hoping that these questions can be addressed in subsequent studies. In addition, as noted by Garvey and Bolton (90), the willingness to purchase ecological products is influenced by various factors. The findings of this study also partly reflect that different types of ecological products may have varying degrees of impact on tourists' green purchasing intentions. Therefore, future research could explore how the interaction between product type and packaging format (transparent vs. opaque) affects tourists' willingness to make green purchases.

## Data availability statement

The raw data supporting the conclusions of this article will be made available by the authors, without undue reservation.

## Ethics statement

The studies involving human participants were reviewed and approved by Ethics Committee of North Sichuan Medical College. Written informed consent from the participants and participants legal guardian was not required to participate in this study in accordance with the national legislation and the institutional requirements.

## Author contributions

TK: Data curation, Investigation, Methodology, Writing – original draft, Writing – review & editing, Supervision. DY: Conceptualization, Data curation, Investigation, Methodology, Project administration, Writing – original draft, Writing – review & editing. DZ: Data curation, Methodology, Resources, Supervision, Validation, Writing – review & editing.
